# Transitioning a multiethnic donor pool from serologic D-negative to molecularly *RHD*-negative at a hospital-based blood donor service

**DOI:** 10.1186/s12967-025-06716-8

**Published:** 2025-06-19

**Authors:** Willy A. Flegel, Kshitij Srivastava, Lorraine G. Caruccio, Pirmin Schmid, David A. Stiles, Marina U. Bueno, Nadine R. Dowling, Traci D. Paige, Sita Shrestha

**Affiliations:** Laboratory Services Section, Department of Transfusion Medicine, NIH Clinical Center, National Institutes of Health, Bethesda, MD 20892 USA

## Abstract

**Background:**

Some individuals carry a very low expression of the D-antigen, called a Del phenotype. Red cell units from such blood donors with *DEL* alleles are RhD protein-positive, despite being routinely labelled D-negative. Molecular typing offers a more sensitive method to identify Del individuals by detecting the presence of the *RHD* gene. Pools of 20 or more donor samples are routinely screened for the *RHD* gene in some, mostly European, donor populations.

**Methods:**

A modular real-time PCR assay targeting *RHD* intron 4, exon 5, and exon 7 was developed for individual testing. We screened for the *RHD* gene among all blood donors who typed D-negative in routine serology.

**Results:**

Over 15 years, 2254 D-negative donors were individually tested for the *RHD* gene. With a sensitivity of detecting 5 *RHD* positive gDNA copies per reaction, 42 donors tested positive (1.9%). Among them, 34 carried the common *RHDΨ* allele (80.9%), while 7 harbored 5 known *RHD* alleles, and 1 a novel *RHD* deletion. We inadvertently detected 2 other donors with DVI, establishing a population frequency of 1 in 731 for the U.S.

**Conclusions:**

A modular approach for *RHD* screening is suitable for blood donors when sample pooling is not feasible among multiethnic donor populations. We transitioned donors since 2009 from serologic D-negative to molecularly *RHD*-negative status at the NIH Clinical Center. Molecular *RHD* screening of serologic D-negative donors is an effective way to identify individuals harboring *DEL* alleles that can cause alloimmunization in transfusion recipients.

**Supplementary Information:**

The online version contains supplementary material available at 10.1186/s12967-025-06716-8.

## Introduction

The D-antigen is the most immunogenic and clinically significant antigen of the Rh blood group system (ISBT 004) [1]. It is expressed on the non-glycosylated RhD protein, encoded by the *RHD* gene located on chromosome 1 [2]. Antibodies targeting the D-antigen can lead to severe hemolytic transfusion reaction (HTR) or hemolytic disease of the fetus and newborn (HDFN) [3]. Donors with a very low expression of the D-antigen (Del phenotype) have been shown to cause both primary and secondary anti-D alloimmunization when transfused to patients lacking the *RHD* gene [4–19].

Several molecular mechanisms are known to cause a D-negative phenotype, with the homozygous deletion of the whole *RHD* gene (RHD*01N.01) being the most common worldwide. Other mechanisms involve an intact *RHD* gene with single nucleotide substitutions, as well as deletions or insertions of one or more nucleotides [20]. Additionally, replacement of *RHD* exons by their highly homologous *RHCE* counterparts can result in hybrid RhD proteins that may or may not express epitopes of the D-antigen [20].

Among serologic D-negative blood donors in Europe, the proportion of the individuals carrying the intact *RHD* gene ranges from 0.2% to 0.4% depending on the geographic region [21]. In non-European populations, the number of serologic D-negative donors with some portion of the *RHD* gene is generally higher and ranges from 10% in Africans (*RHDΨ* and Ccde^s^) [22] to 30% in Asians (Asian-type DEL: RHD*01EL.01, RHD*DEL1, or *RHD:c.1227G* > *A*) [23–26]. Substantial variation in the frequency of different *RHD* alleles among D-negative blood donors across African populations highlights the genetic diversity of the D-antigen on the continent [27–31]. Due to the large African ancestry in the Brazilian population [32], serologic D-negative donors carry an *RHD* allele at rates between those found in African and European populations [33, 34]. D-negative donors from East and Southeast Asia, including China [25], Taiwan [35], Japan [36], Korea [23, 37], Myanmar, [38] Thailand [39] and Malaysia [40, 41] show less variability in *RHD* alleles, with most carrying a Del phenotype encoded by the clinically relevant Asian-type DEL allele [24, 40].

More than 70% of blood donor samples in the U.S. are tested by Creative Testing Solutions (CTS) [42]. The AABB Standards for Blood Banks and Transfusion Services mandate that ‘The Rh type shall be determined for each collection with anti-D reagent. If the initial test with anti-D is negative, the blood shall be tested using a method designed to detect weak D. When either test is positive, the label shall read “Rh POSITIVE” (Sect. 5.8.2) [43, 44]. When the tests for both D and weak D are negative, the label shall read “Rh NEGATIVE”.’ In the U.S. and Europe, D-antigen typing in blood donors is performed using automated systems designed to detect most weak D samples. In Europe, serologic D-negative first-time donors are confirmed using the indirect antiglobulin test (IAT). In some European countries, however, the IAT has been replaced by molecular *RHD* testing, often performed by pooling samples from serologic D-negative donors [45–48]. In the U.S., an IAT is generally not used for confirming serologic D-negative donors.

Systematic *RHD* gene screening in all first-time serologic D-negative blood donors was initially implemented in Germany in 2001, as the first routine red cell genotyping in blood donors [26]. It was adopted by Switzerland in 2012, with mandatory screening beginning in 2013 [48]. Screening using similar or modified molecular protocols has been applied in numerous other institutions [26, 49–56] and commonly involves testing for *RHD* exons 5, 7, or 10, intron 4, or their combinations. Positive samples undergo *RHD* allele characterization using commercial molecular typing kits or nucleotide sequencing.

The objective of this survey was to evaluate the serologic D-negative, multiethnic donor population at the NIH Clinical Center for the presence of the *RHD* gene, without employing sample pooling. Our results demonstrate the practicability of molecular screening using a real-time PCR-based assay for individual testing.

## Materials and methods

### Blood donors

The quality improvement survey included 16,589 routine blood donors at the NIH Blood Center between November 2009 and October 2024. Written informed consent was obtained as part of their blood donation process.

### Automated serologic testing

D-antigen typing of donors was outsourced (CTS at its Phoenix, AZ location until February 17, 2020, and at Tampa, FL since). CTS used an auto-analyzer (PK7300 or PK7400; Beckman Coulter, Brea, California) and 2 anti-D reagents: (i) a monoclonal IgM reagent that does not agglutinate DVI cells [PK1 = clone P3X61 (epD 6.1) or PK2 = clone HM10 (epD 6.6); Diagast, Loos, France]; and (ii) an oligoclonal reagent [IgM clones P3X61, P3X21223B10 (epD 9.1) + IgG clones P3X290 (epD 3.1) and P3X35 (epD 5.4); Diagast] that agglutinates most weak D cells as well as the clinically relevant D category VI (DVI) cells (via P3X21223B10 and P3X290). If both anti-D reagents were negative, no additional test was performed, and the sample was labeled as D-negative.

### Variant testing

If the initial test yields a “No Type Determined” (NTD) result, the sample undergoes additional automated testing (NEO Iris; Immucor, Norcross, Georgia) followed by manual tube testing [57]. On December 18, 2023, CTS in Tampa initiated a pilot study that replaced the standard variant testing algorithm, and applied an automated methods that could not detect some weak D and any DVI cells D (ID-MTS Anti-D Card, clone MS-201; Ortho Clinical Diagnostics, Rochester, NY) [58]. This oversight led to a recall on August 20, 2024, which affected 731 blood donors at the NIH Clinical Center.

### Immunohematology

Additional hemagglutination tests for the D-antigen were performed at NIH by standard tube and anti-IgG gel matrix testing with licensed reagents (Ortho, Raritan, NJ; Bio-Rad Laboratories, Hercules, CA; and Immucor, Norcross, GA, USA). An adsorption/elution method with human polyclonal anti-D was applied to test for the presence of a DEL phenotype [51].

### Molecular screening of D-negative donors

EDTA-whole blood samples collected from blood donors initially classified as serologic D-negative were chosen for subsequent processing. Genomic DNA was isolated from the buffy coat (Qiagen EZ1 DNA blood kit on the BioRobot EZ1; Qiagen, Valencia, CA, USA) [59]. D-negative donor samples were screened individually using 3 real-time PCR assays targeting *RHD* specific DNA sequences in intron 4, exon 5, and exon 7 using primers described previously [51]. The 3 real-time PCR assays were performed in separate reaction tubes (modular) [60] and in triplicates. D-negative donors that had at least one positive real-time PCR result for the detection of the *RHD* gene underwent additional testing to identify *RHD* gene variants using commercial genotyping kits (BAGene, BAG Health Care, Germany; PreciseType HEA, Immucor, USA; and RBC-FluoGene, Inno-train Diagnostik, Germany). The intron 4 real-time PCR assay had an experimentally defined sensitivity, specificity, and linearity.

### RHD real-time PCR assay

We developed a Standard Operating Procedure (Supplementary File S1) and utilized sequence-specific primers (SSP) for *RHD* intron 4 (re41 and rb12), exon 5 (rhd_ex5f and ga51), and exon 7 (ga71 and ga72) [26, 41], in a real-time PCR (CFX96 real-time system and SsoFast EvaGreen Supermix with SYBR green as the intercalating dye; Bio-Rad, Hercules, CA). The 652 bp *RHD* specific deletion in intron 4 [61] represents the most sensitive target in our assay and offers a considerably higher positive predictive value compared to other 2 markers [26].

(i) Specificity: The specificity of the *RHD* intron 4 real-time PCR assay was determined by performing a melting curve analysis after 40 cycles of amplification. The analysis consisted of an automated amplicon melting at a controlled rate of 0.5 °C per 5 s from 65.0 °C to 95.0 °C. Fluorescence data was recorded every 5 s. Melting curves were automatically analyzed (CFX Manager software, version 3.1; Bio-Rad). (ii) Sensitivity: To determine the percent accuracy and limit of detection for the *RHD* intron 4 real-time PCR assay for detecting *RHD* microchimerism, 20 gDNA samples were collected and normalized to 50 ng/µL (10 *RHD* gene-negative and 10 *RHD* gene-positive). These samples were confirmed to be either D-negative or D-positive by serology and *RHD* gene-negative or -positive as described previously [26]. The *RHD* gene copy number per real-time PCR reaction was calculated taking into account that diploid human female nuclei in the G1 phase of the cell cycle contain 6.550 pg of the DNA [62]. A spike-in series was constructed for each sample pair, consisting of 10, 5, 4, 3, 2, and 0 copies of *RHD*-positive gDNA per reaction. Each spike-in point had a background of approximately 15,000 copies of *RHD*-negative gDNA per reaction. Real-time PCR detection was defined by a Cycle quantification (Cq) of ≤ 38 set in the log-linear phase of amplification. (iii) Linearity: A standard curve was created by serially diluting 22,900 copies of gDNA from an RhD-positive donor in molecular biology grade water by a factor of 10 each time for 7 dilution steps. All real-time PCR reactions were run in triplicate, and mean Cq values were calculated for analysis.

### RHD gene sequencing

*RHD* alleles unconfirmed using commercial kits were identified using nucleotide sequencing [63–66]. Zygosity testing for the *RHD* gene was done by restriction fragment length polymorphism (RFLP) [2].

### Red cell unit labeling

The U.S. Food and Drug Administration (FDA) approved in December 2018 (BL 103044/5052) a supplement to our Biologics License Application (BLA): “to label red blood cells as Rh positive from DEL phenotype donors who test D-negative by licensed serological blood grouping assays, but genotype as RhD-positive using your laboratory-developed and validated molecular assay specific for the *RHD* gene Intron 4, Exon 5, and Exon 7”.

## Results

**Fig. 1:**
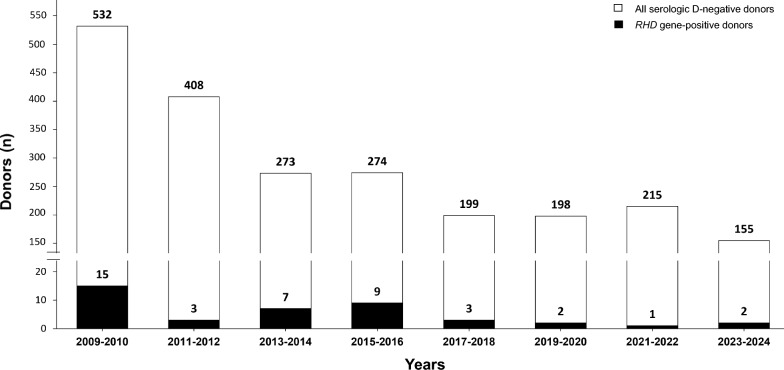
All serologic D-negative donors and the *RHD* gene-positive donors over 15 years. A total of 2254 serologic D-negative donors (□) were screened between November 2009 and October 2024. Among them, 42 donors tested positive for the *RHD* gene (■). Inadvertently, 2 additional donors with a DVI phenotype were found (not shown)

A molecular *RHD* screening of all serologic D-negative donors was carried out at the NIH Clinical Center over a 15-year period. Each donor was tested once at first encounter after the start of the program in 2009. Due to the diversity of *RHD* alleles in African and Asian populations [67], and the substantial representation of African American and East-Asian American blood donors in our community, we opted for a modular real-time PCR assay instead of a pooled assay. Our screening utilized three real-time PCR assays targeting *RHD*-specific sequences in intron 4, exon 5, and exon 7. Among the 2254 D-negative donors screened, 42 tested positive (1.86%) in one or more of the PCR assays (Table 1). The number of whole blood donors who needed the molecular testing declined rapidly over time (Fig. 1).

**Table 1:** Genotyping for *RHD* intron 4, exon 5, and exon 7 in 2254 serologic D-negative whole blood, plasma, platelets, and white blood cell donors

Characteristic	Serologic D-negative donors^a^			
	Total (n)	*RHD* gene-positive^b^		
		n	%	*p*^e^
Female	1286	26	2.02	0.637
Male	968	16	1.65	
Total	2254	42	1.86	
Race/ethnicity				
African American	121	31	25.62	< 0.00001
White	1889	4	0.21	
Asian	44	2	4.54	
Hispanic	60	2	3.33	
American Indian	2	0	n.a	
Unknown^c^	138	3	2.17	
Total	2254	42	1.86	
Rh phenotype				
ccee	1914	25	1.31	< 0.00001
Ccee	74	9	12.16	
CCee	3	1	33.33	
ccEe	38	0	n.a	
Not tested^d^	225	7	28.00	
Total	2254	42	1.86	

n.a., not applicable

^a^CTS result by routine donor screening methods not involving adsorption/elution

^b^*RHD* gene-negative or -positive by real-time PCR for *RHD* intron 4, exon 5, and exon 7

^c^Response declined or no response in the blood donor questionnaire

^d^Platelet, white blood cells and plasma donors who were not phenotyped for RhCE antigens

^e^Fisher’s exact test, two-tailed

### Red cell unit labeling

Since 2009, the NIH Blood Bank has been tagging Asian-type DEL red cell units as DELPH (Del phenotype—RH positive patient only). Following an FDA approval of our BLA supplement in 2018, we started labeling DEL red cell units as Rh positive.

### *RHD* intron 4 real-time PCR development

**Fig. 2:**
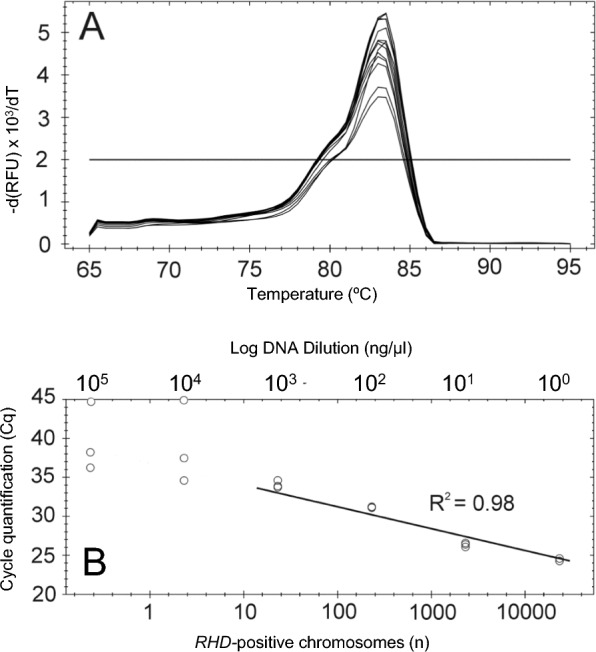
*RHD* intron 4 real-time PCR assay. **A** *RHD* intron 4 real-time PCR melting curves have a single peak at 83.2 °C for a double-stranded PCR amplicon. The horizontal line represents the instrument’s default setting for the threshold of positive results. **B** Real-time standard curve of *RHD* intron 4 displaying the relationship between Cq and *RHD*-positive chromosome copies. The circles represent RhD-positive genomic DNA standards along the curve. The 2 standard points at Cq = 45 are outside of the linear dynamic range of the assay

(i) Sensitivity: The real-time PCR assay demonstrated a 100% detection rate for *RHD* gene-positive and *RHD* gene-negative sample pairs, with 10 and 5 *RHD* positive gDNA copies per reaction (Table 2). The detection rate was less than 100% when fewer than 5 *RHD* positive gDNA copies were added to the real-time PCR reaction. (ii) Specificity: The specificity of the real-time assay primers was confirmed by the consistent dissociation temperature observed for the double-stranded amplicon. The mean dissociation temperature across all positive PCR reactions was 83.2 °C (Fig. 2A). (iii) Linearity: We determined experimentally a linear dynamic range from 22.9 to 22,900 *RHD* gene copies per microliter. The mean amplification efficiency across 3 experiments was 88.1% and the mean R^2^ was 0.98 (Fig. 2B). Any target concentration below 22.9 *RHD* gene copies per microliter was outside of the range of detection for the assay and yielded inconsistent, non-linear results (Fig. 2B).

**Table 2:** Real-time PCR detection of *RHD* gene-positive gDNA in a background of *RHD* gene-negative gDNA

	Real-time PCR				
*RHD* positive chromosomes (n)	Genomic DNA (ng) per reaction		Positive results in 10 reactions (n)		
	*RHD* positive	*RHD* negative	Observed^a^	Expected	Rate of positive results
10	0.0655	98.25^b^	10	10	100%
5	0.03275	98.25	10	10	100%
4	0.0262	98.25	9	10	90%
3	0.01965	98.25	4	10	40%
2	0.0131	98.25	4	10	40%
0^c^	0	98.25	0	0	0%
0^d^	0	0	0	0	n.a

n.a., not applicable

^a^Cq < 38.0, test in triplicate (mean)

^b^98.25 ng gDNA represent 15,000 copies of *RHD* negative gDNA

^c^No template control

^d^Water control (no DNA)

### Blood donors with *RHD* alleles

Among the 42 *RHD* gene-positive donors, 34 harbored the common *RHDΨ* allele (80.95%), typically in individuals with African ancestry (Table 3). Among the remaining 8 donors, 7 carried 5 known *RHD* alleles, while 1 donor carried an allele (*RHDex1del type 1*), that was novel at the time [59]. Only 5 individuals were found (0.22%) among our D-negative blood donors, who required labelling as Rh positive per FDA approval. We inadvertently found 2 other donors with a DVI phenotype who required labelling as Rh POSITIVE per AABB standards [43, 44]. However, among the 2254 donors in 15 years, who resulted as D-negative in the automated serologic testing by an autoanalyzer, we found only 1 sample with a weak D type allele (Table 3).

**Table 3:** *RHD* alleles identified in 42 *RHD* gene-positive serologic D-negative donors^a^

Allele			Samples (n)	Real-Time PCR Result			Serology			Self-declared ethnicity of donors (n)	Red cell units labelled	Reference
Trivial name	ISBT allele no	GenBank no		Intron 4	Exon 5	Exon 7	D	Adsorption/elution	CcEe			
*RHDΨ*	RHD*08N.01	MN783007	34	+	–	+	D-negative	not performed	ccee ccee ccee Ccee Ccee unknown unknown unknown	19 African American 2 Hispanic 2 unknown 2 African American 2 African American^b^ 5 African American 1 White 1 unknown	D-negative	[22]
*RHD(1227G* > *A)*	RHD*01EL.01	JQ424879	3	+	+	+	DEL	positive	Ccee Ccee CCee	1 White 1 Asian 1 Asian^d^	D-positive^c^	[26]
*RHDex1del type 1*	RHD*01N.67	KX584097	1	+	+	+	DEL	positive	ccee	African American	D-positive^c^	[59]
*Weak D type 4.0*^e^	RHD*09.03.01	KR260900	1	+	+	+	D-negative	negative	ccee	African American	D-negative	[63]
*RHDex10del type 1*	Not available	KX584099	1	+	+	+	DEL	positive	Ccee	White	D-positive^c^	[80]
*RHD(487delAGAC)*	RHD*01N.13	AF037626	1	+	+	+	D-negative	negative	Ccee	White	D-negative	[82]
*RHD(330delGT)*^f^	RHD*01N.35	EF105440	1	+	+	+	D-negative	negative	Ccee	African American	D-negative	[83]
Total			42									

^a^Excluding the inadvertently found 2 blood donors with DVI phenotype (see text in the Results section)

^b^Both donors observed *in trans* with *(C)ce*^*s*^* type 1* haplotype, RHD*03N.01, *KF515558* [84]

^c^5 individuals who donated 45 red cell units during the study period

^d^Possibly homozygous for RHD*01EL.01 allele; zygosity not tested

^e^Molecular cause for serologic D-negative phenotype unknown

^f^Observed in trans with (C)ce^s^ type 1 allele, RHD*03N.01, KF515558 [84]

### Donor with weak D type 4.0 (RHD*09.03.01)

We identified an individual with a hemizygous *weak D type 4.0* allele (Table 3). Serologic testing for weak D, additional blood group antigens, and adsorption/elution consistently confirmed the D-negative result, ruling out sample contamination or mix-up, and also excluded chimerism (data not shown). However, sequencing of the *RHD* and *RHCE* genes from genomic DNA, along with direct PCR and sequencing from frozen packed red cells, revealed no explanation for the D-negative phenotype. Further investigation would be needed to discern the molecular basis in this sample that was properly labeled serologic D-negative.

### Donors with DVI (RHD*06)

In addition to the 2254 D-negative donors included in this survey (Table 1), 2 other donors were reported as D-negative by CTS. An *RHD* gene was detected in these individuals who both were found to carry a DVI type 2 (RHD*06.02) allele (Table 4). DVI were not expected among our serologic D-negative donors but were inadvertently found due to the presence of a C antigen *in trans* (2018 sample) and incorrect serologic donor typing (2024 sample). These unexpected observations allowed us to calculate a DVI frequency of 1:731 in our donor population (Table 4) [68], which was higher than the frequencies reported elsewhere, such as 1:6,214 in Germany [69].

**Table 4:** Frequency of *DVI type 2* in the study

Donation	Cohort size	Time frame	Ethnicity	Rh phenotype	*RH* haplotype *in trans*	*RHD* Zygosity	Nomenclature		Reason for observation	Frequency		
							Trivial	ISBT		Observed in study	Occurrence in population	
											Corrected	95% CI
July 2018	16,589	Nov 2009 – Oct 2024	White	CCDee	*Cde*	Hemizygous	*DVI type 2*	RHD*06.02	C *in trans* [77, 78]	1:16,589	1:565^a,b^	1:106–1:11,078
February 2024	731	Dec 2023—Aug 2024	White	CcDee	*cde*	Hemizygous	*DVI type 2*	RHD*06.02	Incorrrect typing [8]	1:731	1:731^b^	1:137–1:14,333

^a^Corrected for *RH* haplotype frequency in the U.S. [68] = 1:16,589*(frequency of *cde* + frequency of *cdE*)/(frequency of *Cde*) = 1:16,589*(36.09 + 0.61)/1.25 = 1:16,589*29.36 = 1:565

^b^Not statistically significant when compared with DVI frequency in Germany (1:6,214; Fisher's exact test P ranging from 0.094 to 0.119) [69]

95% CI, 95% confidence interval (Poisson distribution, two-tailed)

### Worldwide prevalence of *RHD* among D-negative donors

The prevalence of *RHD* gene among serologic D-negative donors varies widely across different ethnic groups [70]. Our 15-year survey corroborates the findings described in 58 studies since the late 1990 s (Table 5 and Supplementary Table S1), documenting Europeans (Whites) having the lowest prevalence (< 1%) of *RHD* gene-positive individuals among serologic D-negative donors, and Sub-Saharan Africa (61%) and East Asia having the highest prevalences (28%).

**Table 5:** Molecular screening of serologic D-negative donors for the *RHD* gene in 58 publications^a^ and this study

		Serologic D-negative donors						
		C +/E + individuals			cde individuals			
		*RHD* gene-positive		All	*RHD* gene-positive		All	Total (n)
		n	%		n	%		
Europe	10 countries^b^	383	3.67	10,444	936	0.35	255,124	265,568
North America	Canada, United States^c^	15	7.65	196	103	1.39	7201	7397
South America	Argentina, Brazil	309	26.43	1,169	321	4.97	5314	6,463
Northern Africa	Tunisia	42	22.95	183	26	2.43	888	1,071
Sub-Saharan Africa	Congo	n.a	n.a	n.a	104	61.54	169	169
East Asia	China, Japan, Korea^d^	1791	56.55	3167	2022	28.39	3934	7121
Middle East Asia	Iran, Saudi Arabia	2	12.5	20	4	1.00	446	466
Southeast Asia	Thailand	355	50.9	698	61	6.3	963	1661
South Asia	India	57	15.8	361	1	10	10	371
Total		2954	n.a	16,238	3578	n.a	274,049	290,287

^a^For a list of the 58 publications see Supplementary (Table S1)

^b^Austria, Denmark, Germany, Italy, Netherlands, Poland, Slovenia, Switzerland, Bosnia and Herzegovina, Russia

^c^Including data from the current study (2254 of 7397 donors)

^d^Including 4 studies from Taiwan (296 of 1791 donors)

n.a., not available

## Discussion

With an average 15% D-negative rate [71], an estimated 1 million D-negative individuals contribute approximately 1.8 million red cell units in the U.S. each year [72, 73]. Even rare errors in accurately identifying a blood group phenotype when relying on serology alone can result in many red cell units failing to meet AABB standards [43, 44] or best practice in unit labeling. Red cell genotyping is more sensitive than any serologic method [55, 74, 75]. To overcome the limitations of relying on serology alone, routine molecular *RHD* screening for serologic D-negative donors has originally been implemented in 2001 [26] and in many European institutions since (Table 5) [50–55]. We established a method for *RHD* screening applicable to any population, exploring the impact of identifying *RHD* alleles in multiethnic donor cohorts. We screened 2254 D-negative donors in 15 years at the NIH Clinical Center. This cohort represents the turnover of large blood centers in a week or even a couple of days, indicating the scope of novel or unexpected observations (Table 3) still attainable after 25 years of study.

We observed a decrease in the number of tests over time, which eventually stabilized at a baseline, as expected for any newly introduced analyte (Fig. 1) and reported previously [26]. The higher rate of *RHD* gene-positive donors between 2013 and 2016 was attributed to the *RHDΨ* [22] allele among African Americans, whom we actively recruited during this period. If we had aimed for a gradual implementation with a constant number of tests each year, we could have tested, for instance, first-time donors [51]. After a few years of the program, most tests were required in first-time donors only. Based on the required proper donor documentation, each donor needs to be tested only once per lifetime, making red cell genotyping a highly cost-efficient approach to a remarkable degree.

Our red cell genotyping assay for D-negative blood donors identified 5 *RHD* gene-positive individuals with a DEL phenotype, confirmed by adsorption/elution, who, in accordance with our FDA approval, should be labeled as Rh positive. These 5 individuals donated a total of 45 red cell units, all of which would have been labeled D-negative, as serology missed their DEL phenotype. Assuming similar population frequencies throughout the country, this rate would amount to approximately 3,960 DEL red cell units (0.22% × 1.8 million) currently labeled as D-negative in the U.S. per year [24]. The DEL red cells caused by 2 alleles in 4 of our donors (*RHD(1227G* > *A)* and *RHDex10del type 1*;Table 3) are known to have induced primary [5–9, 11–18] and secondary anti-D immunizations [4, 10, 19].

If the serologic typing had missed samples with weak D phenotypes, we would have expected to find approximately 20 cases in our cohort, assuming a maximum frequency of 1% for the most common weak D phenotypes (types 1, 2, and 3) [76]. Despite being performed without IAT, the automated serologic testing successfully detected all weak D alleles. This observation underscored the effectiveness of the technical approach that is commonly used by blood centers in the U.S. The unexpected finding of 2 samples with the DVI phenotype highlighted the strength of our study as a quality assurance measure in 2 ways: First, the DVI sample observed in 2018 exhibited a CCee phenotype (Table 4) with its C antigen *in trans* exerting a suppressive effect on the D antigen density [77, 78]. This effect, along with the known limitations of the reagents in detecting some rare, weak, or variant D phenotypes [79], explained the missed detection during automated serologic testing. Second, the DVI sample from 2024 exhibited a Ccee phenotype (Table 4) and belonged to a group of 731 blood donors whose serologic D-antigen test results were deemed invalid and recalled (see “Materials and Methods” section). The DVI frequency at NIH (1:731) differed markedly from Germany (1:6,214) without reaching statistical significance (*p* = 0.119, Fisher’s exact test). As our cohort was small, limiting its statistical power, further studies with larger samples in the U.S. will determine if a difference exists.

A novel *RHD* allele with a DEL phenotype was identified in an African American donor (*RHDex1del type 1*; Table 3) [59]. Additionally, another *RHD* allele with a DEL phenotype was found in a White donor (*RHDex10del type*). This latter allele was first reported in France in 2012 [80] and has been linked to secondary anti-D immunization in an *RHD* gene-negative transfusion recipient who had received a single red cell unit [19]. The breakpoints of both alleles with deletions had been published [81]. We also observed a donor harboring a rare 4-nucleotide deletion in *RHD* exon 4 [*RHD(487delAGAC*), Table 3] [82] and another donor heterozygous for 2 different *RHD* alleles: *RHD*(330delGT) [83] and *(C)ces type 1* [84].

Our modular real-time PCR based screening strategy for individual D-negative donor testing detects the *RHD*-specific DNA sequences located in intron 4, exon 5, and exon 7. Our approach is effective for populations of African ancestry, as the lack of amplification in exon 5 serves as a surrogate marker for the *RHDΨ* allele [85]. It is also particularly useful for Asian populations, where the incidence of RHD*DEL1 allele can reach up to 30% among D-negative individuals [23–26, 70, 86]. We performed individual testing due to the small number of donors at our hospital-based blood donor service center. However, our approach can be implemented as pooled testing at large U.S. blood centers. For example, pooling 20 [50, 55, 56] or 24 samples [56] from serologic D-negative donors may enable a rapid and more cost-effective molecular screening of D-negative donors.

Adding molecular *RHD* screening for serologic D-negative donors will improve the safety of red cell units and reduce primary and secondary anti-D immunizations of patients where serologic testing is the only test applied in the U.S [44]. Replacing the IAT by molecular *RHD* screening is an approach to detect all Del phenotypes, along with weak D or partial D [87, 88]. There is no good excuse for relying on IAT, which is known to yield incorrect results in clinically relevant instances, when IAT can be replaced by modern technology at similar or lower cost [89]. Red cell genotyping for the *RHD* gene proved to be a practical method for large-scale screening of *RHD* variants in many settings worldwide with routine experience over more than 2 decades. The approach using a real-time PCR-SSP method resolves the known limitations of serologic testing, thereby avoiding anti-D immunizations and reducing the risk of their adverse clinical consequences.

## Supplementary Information

Additional file 1. **Supplementary File S1.** Standard Operating Procedurefor the *RHD* genomic screening of D-negative blood donors. The excerpt from our SOP documents the process utilizing real-time PCR-SSP for *RHD* Intron 4, Exon 5, and Exon 7 to accurately identify the presence of the *RHD* gene.

Additional file 2. **Supplementary Table S1.** Molecular screening of serologic D-negative donors for the *RHD* gene in 58 studies. A complete list of publications from 2001 to 2024 has been compiled documenting the types of *RHD* screening and the total number of donors screened worldwide in clinical studies over the past 25 years.

## Data Availability

Data will be made available on reasonable request.
