# Supplementary material for: Transitioning a multiethnic donor pool from serologic D-negative to molecularly RHD-negative at a hospital-based blood donor service
Source: J Transl Med. 2025 Jun 19;23:686. doi: 10.1186/s12967-025-06716-8 (PMC12180192; doi:10.1186/s12967-025-06716-8)
Supplement: Supplementary file 1 — Additional file 1. Supplementary File S1. Standard Operating Procedurefor the RHD genomic screening of D-negative blood donors. The excerpt from our SOP documents the process utilizing real-time PCR-SSP for RHD Intron 4, Exon 5, and Exon 7 to accurately identify the presence of the RHD gene. [file 12967_2025_6716_MOESM1_ESM.pdf]

RHD genomic screening of D-negative blood donors by real-time PCR

1. PRINCIPLE OR PURPOSE

The purpose is to screen at the genomic level using a real time PCR method, the *RHD* Intron 4, Exon 5, and Exon 7 status of NIH donors who are D-negative by serologic testing methods. To determine the nature of the alleles and if the *RHD* genotype is positive in these donors, refer to references listed at the end of this SOP for more detail.

If the real-time PCR *RHD* Intron 4, Exon 5, and Exon 7 genotyping reactions are negative then no further testing is needed with the real-time PCR platform. If they are positive, then additional testing is performed to identify the allele. If there are weak signal positives, the reactions will be repeated. Determination of the final interpretation of D type is made by the Chief, Laboratory Services after review of all testing.

Real-time PCR screening of donor samples uses primers specific for the *RHD* gene Intron 4, Exon 5, and Exon 7 regions. The three assays detect essentially all *RHD* gene positive, serologic negative samples, which can express the D antigen. No internal control is used with these assays and all samples are tested in triplicate. Using the conditions outlined in this SOP the *RHD* Intron 4, Exon 5, and Exon 7 real-time PCR assays should not amplify analogous regions in the *RHCE* gene.

2. SCOPE

The scope of this SOP includes testing donor gDNA samples using a real-time PCR assay, analyzing and recording results, and determining if further testing is warranted.

3. KEY WORDS

Genotyping, *RHD*, *RHCE*, Allele, real-time PCR.
Ct = Cycle threshold
Cq = Cycle quantity
Ct and Cq are synonymous terms. DTM uses Ct.
RFU = Relative Fluorescence Units
NTC = No Template Control

4. RESPONSIBILITIES

This method must be performed by qualified staff trained and deemed competent by a qualified trainer.

All *RHD* genotyping results must have an independent second review performed and documented by qualified staff. All additional testing must also be reviewed and documented by qualified staff. Questions concerning anaylsis or validity of a run should be directed to the research support technologist.

External reports on *RHD* genotyping including supplementary tests, must be reviewed and signed by the Chief, Laboratory Services Section or designee.

5. SPECIMEN

- Samples from donors identified as D-negative.
    - Requirements:
        - Purified leukocyte genomic DNA (gDNA) preferrably from EDTA whole blood
    - Segments from leukoreduced donor units are not acceptable
- Sample storage
    - Store at -20 ^{o}C before and after short term use.
    - Long term storage of samples, greater than six months, may be stored at -65 °C or below.

6. EQUIPMENT AND REAGENTS

BIO-RAD C1000 or C1000 Touch Thermal Cyclers
BIO-RAD CFX96 1CFX Connect Real-Time System
CFX Manager Software Version 3.1
Sorvall Legend T Centrifuge
Vortex
PCR grade water
0.2 mL PCR 8-Strip Tubes
Pipettes and PCR grade sterile aerosol-filter low retention pipette tips
Eppendorf style 1.5mL sterile, PCR grade tubes
BIO-RAD Low 96-well White PCR Plates
BIO-RAD Microseal 'B' Film PCR Seals
*BIO-RAD SsoFast EvaGreen Reagent**
*Primers specified in section 11.2**

7. MAINTENANCE

Thermal cyclers used in the performance of clinical tests are verified biannual and records of maitenance maintained in EqM (Equiptment Management).

8. QUALITY CONTROL

The quality of the real-time PCR reactions are partially determined by the uniformity of the dissociation temperature of the double stranded amplicon determined during the melt curve analysis at the conclusion of all real-time PCR runs. A dissociation temperature is based upon the specific size of the amplicon for each positive reaction. The use of gDNA that has been previously genotyped as *RHD* gene positive and gene negative may be used as real-time PCR assay controls. In addition, a water, no template control (NTC), consisting of all reaction components except gDNA should be run as well. This NTC along with the *RHD* gene negative control should be negative.

Troubleshooting:

- Use only in date reagents, kits and supplies.
- Gently vortex and centrifuge the prepared master mix before use.

- Aliquot all reagents and primers and avoid repeated freeze-thawing.
- It is possible for the primers to be amplified as primer-dimers visible on the melt curve plot as a short peak at 75 ^{o}C.
- Very tall primer-dimer peak in an established real-time PCR reaction can indicate that the primers are degraded and should be replaced with fresh primer aliquots.

9. PROCEDURE

9.1. Set up *RHD* screening real-time PCR reactions for Intron 4, Exon 5 and Exon 7.
- Note: Intron 4*,* Exon 5 and Exon 7 are set up as three separate reactions.
- Each different real-time PCR reaction uses two primers. See primer lists 1, 2, and 3 for more information in section 10.4

9.2. The following table is a general scheme for set up of a single reaction; all volumes are in μL. All concentrations for reagents are for the stock. The set up for more than one reaction is scaled up as needed. Thaw all reagents completely before using.

Sample 1 Rxn	Total Volume	PCR grade H_2O	*Forward* Primer (10μM)	Reverse Primer (10 μM)	gDNA	SsoFast EvaGreen Reagent (2x)
W0921-	20	7	1	1	1	10

9.3. Example (20 total reactions): Always add three additional reactions for scaled up volumes to account for pipetting errors (see below). A master mix is made, one for each unique gDNA sample and primer set combination to be tested, including *RHD* gene positive and gene negative control sample (concentration ≥ 50 ng/μL) and NTC which are also run in triplicate.
- The total volume of 20μL is distributed to each reaction well of the BIO-RAD Low 96-well White PCR Plate.
- Remember that for single or scaled up reactions the total volume of gDNA sample and water is always 8μL.
- All primer pair mixes are made of equal volumes of equal concentrations of 10μM primers to yield a final working concentration of 500 nM in each real-time PCR reaction.
- Once the BIO-RAD Low 96-well White PCR plate is loaded and sealed with BIO-RAD Microseal ‘B’ Film PCR Seals, centrifuge in the Sorvall Legend T Centrifuge located in the pre-PCR lab for 2 minutes at 1,260 RPM to collect all transferred liquids to the bottom of the PCR plate and remove any air bubbles.

20 Rxn	Total Volume	PCR grade H_2O	*Forward* Primer (10μM)	Reverse Primer (10 μM)	gDNA	SsoFast EvaGreen Reagent (2x)
20 Rxn	460	161	23	23	23	230

9.4. Primer list 1: Used for detection of *RHD* Intron4 specific real-time PCR reactions. Two primers are used for each sample.

Primer Names	Orientation	Primer Sequences	Product Size bp

re41	Forward	5'- CGA TAC CCA GTT TGT CTG CCA TGC -3'	224
rb12	Reverse	5'- TCC TGA ACC TGC TCT GTG AAG TGC -3'	

Primer list 2: Used for detection of *RHD* Exon5 specific real-time PCR reactions. Two primers are used in each reaction for each sample.

**Primer Names**	**Orientation**	**Primer Sequences**	**Product Size bp**
rhd_ex5f	Forward	5'- CTT CTT GTG GAT GTT CTG GCC AAG TT -3'	168
ga51	Reverse	5'- CTG CTC ACC TTG CTG ATC TTC CC -3'	

Primer list 3: Used for detection of *RHD* Exon7 specific real-time PCR reactions. Two primers are used in each reaction for each sample.

**Primer Names**	**Orientation**	**Primer Sequences**	**Product Size bp**
ga71	Forward	5'- GTT GTA ACC GAG TGC TGG GGA TTC -3'	123
ga72	Reverse	5'- TGC CGG CTC CGA CGG TAT C -3'	

9.5. Set up of the real-time PCR Instrument.

The following is a general scheme for using the Thermal Cycler CFX96 Real-Time System with the BIO-RAD C1000 or C1000 Touch Thermal Cycler instrument to perform real-time PCR using BIO-RAD CFX Manager Software version 3.1 and BIO-RAD's SsoFast EvaGreen Supermix reagent.

Turn on the BIO-RAD C1000 or C1000 Touch Thermal Cycler instrument using the switch in the back. The instrument does a quick Self Test. When complete the screen will say Self Test Complete.

For the *RHD* Intron4 genomic screening of D-negative blood donors use the following thermal cycler protocol:

Step Number:	Temperature (ºC)	Time (Min)	Notes
1	98.0	2:00	
2	98.0	0:05	
3	65.0	0:10	Go to Step 2, 39 more times
4	65.0 to 95.0	0:05	Increase at a rate of 0.5 ºC at 0.5 sec intervals.

For the *RHD* Exon 5 genomic screening of D-negative blood donors use the following thermal cycler protocol:

Step Number:	Temperature (ºC)	Time (Min)	Notes
1	98.0	2:00	
2	98.0	0:05	
3	62.0	0:05	Go to Step 2, 39 more times
4	65.0 to 95.0	0:05	Increase at a rate of 0.5 ºC at 0.5 sec intervals.

For the *RHD* Exon 7 genomic screening of D-negative blood donors use the following thermal cycler protocol:

Step Number:	Temperature (ºC)	Time (Min)	Notes
1	98.0	2:00	
2	98.0	0:05	
3	64.4	0:05	Go to Step 2, 39 more times
4	65.0 to 95.0	0:05	Increase at a rate of 0.5 ºC at 0.5 sec intervals.

9.6. Visualize results with CFX Manager Software version 3.1.

10. ACCEPTANCE CRITERIA

The BIO-RAD CFX Manager Software version 3.1 determines the cutoff threshold, melt temperatures and standard deviations. Final acceptance of results is determined by individual analysis of the run data.

11. INTERPRETATION OF RESULTS

Open up the quantitation data, the melt curve, and melt peak. Verify that the data is acceptable using the following parameters:

- The cutoff threshold value determined by the software must be within the log phase of the assay (Figure 1).

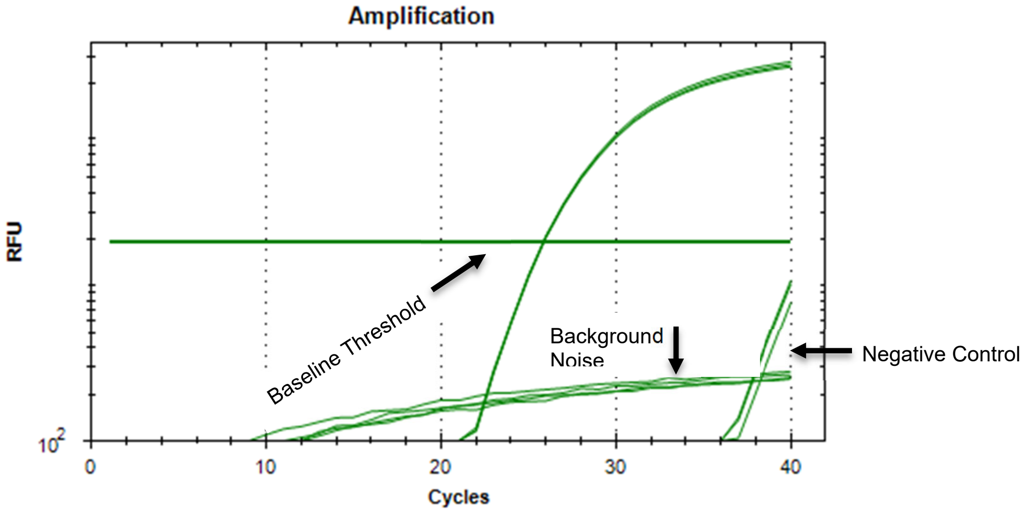

  - Figure 1: Example of an acceptable log phase software assigned cutoff threshold from the BIO-RAD CFX Manager Software version 3.1.

- The NTC must be negative. If there is amplification in the NTC above the negative control the run is invalid (Figure 2). A small amount of primer-dimer amplification may show with the NTC as a small peak at about 75ºC on the melt peak.

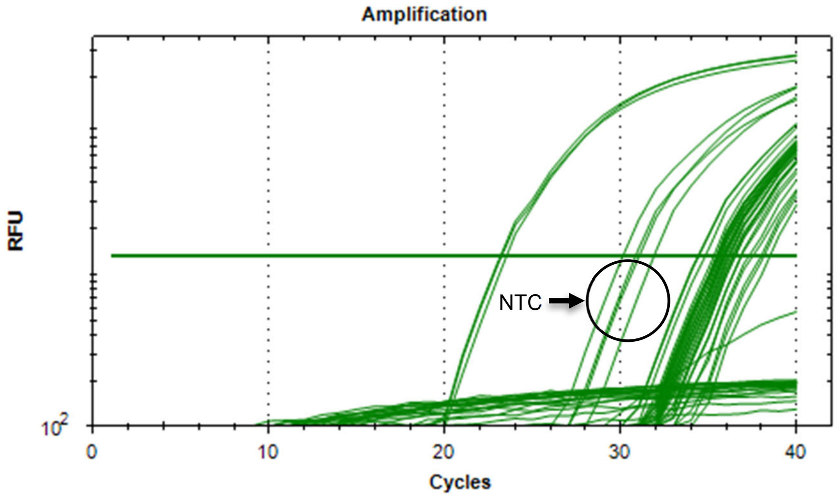

- Figure 2: Example of unacceptable amplification in the NTC from the BIO-RAD CFX Manager Software version 3.1.

- Unknown positive samples must be within +/- 2 Ct values of the positive control (Figure 3).

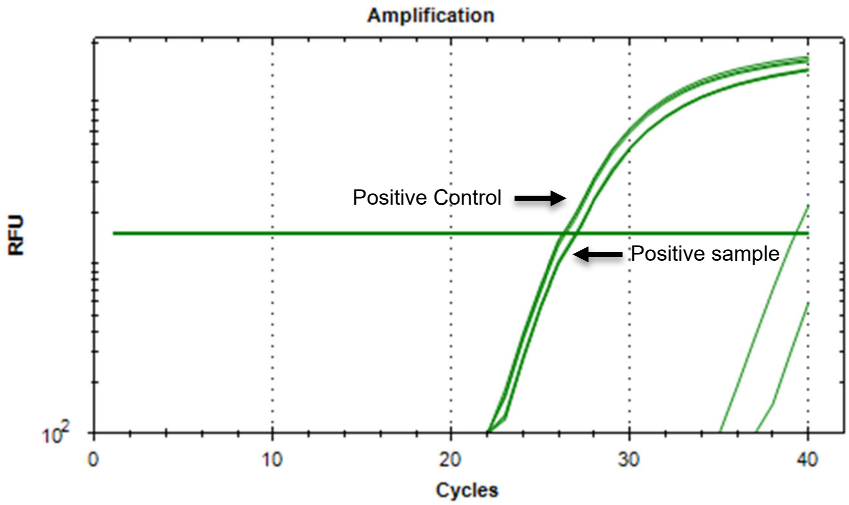

- Figure 3: Example of an acceptable result for an unknown positive sample within 2 Ct values of the positive control from the BIO-RAD CFX Manager Software version 3.1.

- Questionable or weak signal results must be repeated (Figure 4).

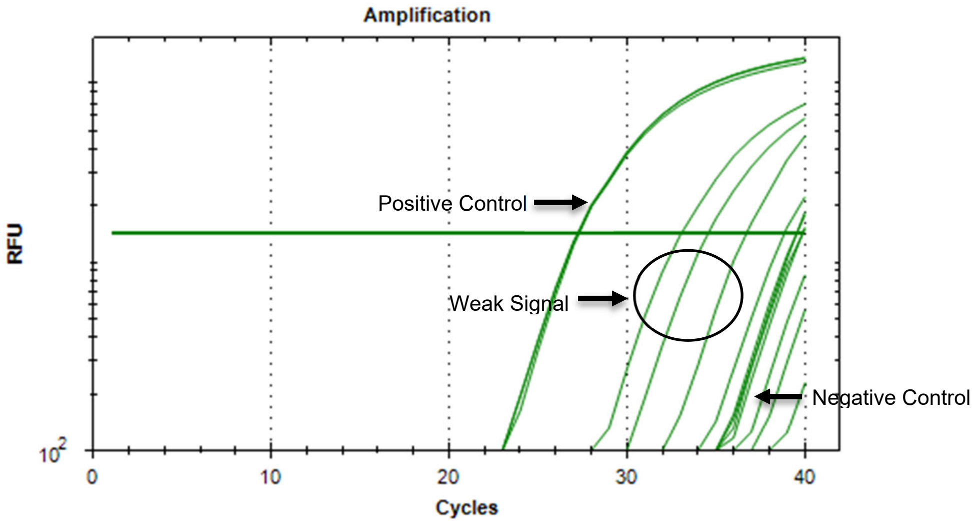

- Figure 4: Example of a triplicate sample of weak signal results to be repeated from the BIO-RAD CFX Manager Software version 3.1.

- The standard deviation for the triplicates of the positive samples and positive controls must be less than 0.500.

- Melt Curve Analysis:
  - Note: “Approximately” is defined as +/- 2°C of the expected melt temperature.
  - A small shoulder on the left side of the melt peak about midway up is acceptable as this is often seen with gDNA as starting template and seems particular to the amplicon of the *RHD* gene with these conditions.
  - Intron 4 Assay: Positive controls and positive samples must have a value of approximately 83.0 °C and have only a single peak, corresponding to a single amplicon (Figure 5).

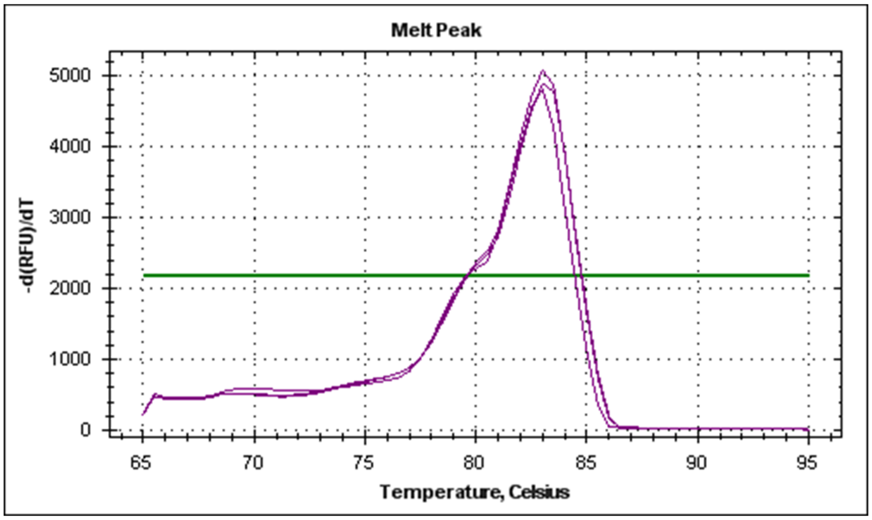

- Figure 5: Example melt peak plot from the BIO-RAD CFX Manager Software version 3.1 for a RHD gene positive sample.

- Exon 5 Assay: Positive controls and positive samples must have a value of approximately 83.5 °C and have only a single peak, corresponding to a single amplicon (Figure 6).

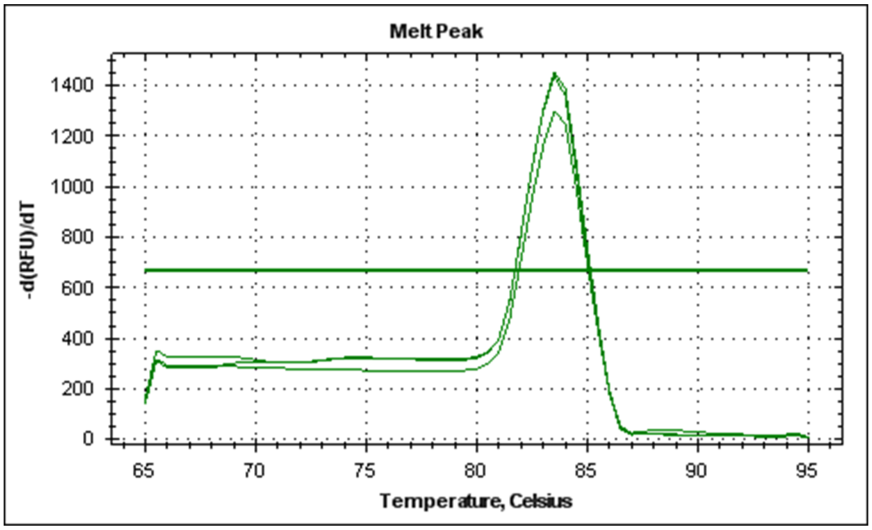

- Figure 6: Example melt peak plot from the BIO-RAD CFX Manager Software version 3.1 for a RHD gene positive sample.

- Exon 7 Assay: Positive controls and positive samples must have a value of approximately 84.0 ºC and have only a single peak, corresponding to a single amplicon (Figure 7).

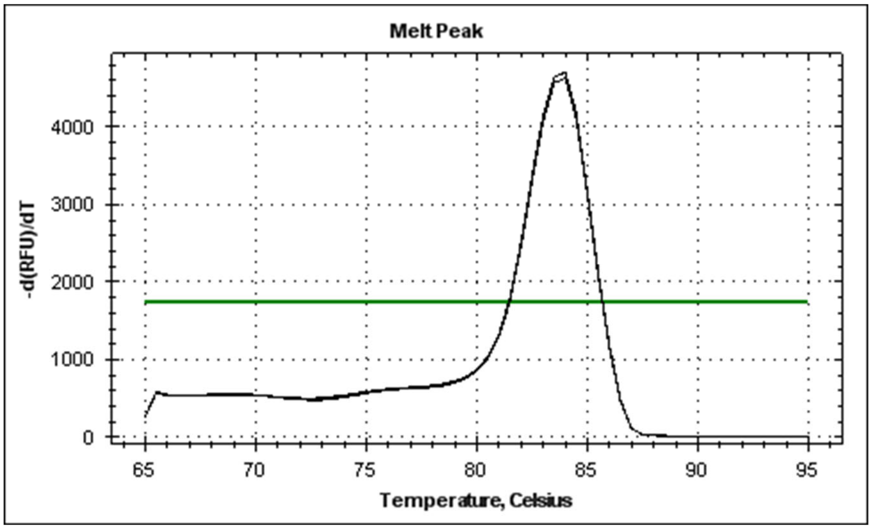

- Figure 7: Example melt peak plot from the BIO-RAD CFX Manager Software version 3.1 for a RHD gene positive sample.

- Negative controls must not have a melt peak above the threshold calculated by the CFX Manager Software version 3.1.

Analyze results using the BIO-RAD CFX Manager Software version 3.1.
Interpretation of results is done independently by two people. Usually the person running the assay is the first person to analyze results.

- Results that are clearly positive or negative as indicated by the expected Ct value and melt peak should be reported as such.
- The standard deviation for the positive samples must be less than 0.500. If the standard deviation is greater than 0.500 the assay is repeated due to inconsistency.

- Samples are run in triplicate to assist in result analysis. Two of three should meet the expected criteria to make an interpretation. Inconclusive or questionable results should be repeated.
- Results that are inconclusive or inconsistent should be repeated in triplicate with the same gDNA sample vortexed for 10 seconds and centrifuged for 30 seconds with a fresh lot of real-time PCR assay specific primers used.
- If results are still inconclusive or inconsistent then a new gDNA sample should be tested when available.
- The sample that is in house can be sequenced with appropriate primers to resolve the results as directed by the Chief, Laboratory Services Section.

12. PROCEDURAL NOTES/SPECIAL HANDLING/SAFETY REQUIREMENTS

Always observe universal precautions for handling potentially infectious material. Wear gloves and disposable labcoats during all procedures.

13. LIMITATIONS OF PROCEDURE

Only Intron 4, Exon 5, and Exon 7 are tested with real-time PCR assays at this time.
Sequencing is required if a new allele is suspected.

14. REFERENCES

a. Flegel WA, von Zabern I, Wagner FF. Six years' experience performing RHD genotyping to confirm D- red blood cell units in Germany for preventing anti-D immunizations, Transfusion 2009;49:465-71.

b. Wagner FF, Gassner C, Muller TH, Schonitzer D, Schunter F, Flegel WA. Molecular Basis of weak D phenotypes. Blood 1999;93:385-93.

c. Flegel WA, von Zabern I, Doescher A, Wagner FF, Strathmann KP, Geisen C, Palfi M, Pisacka M, Poole J, Polin H, Gabriel C, Avent ND. D variants at the RHD vestibule in the weak D type 4 and Eurasian D clusters. Transfusion 2009;49:1059-69.

d. Flegel WA, Wagner FF, Muller TH, Gassner C. Rh phenotype prediction by DNA typing and its application to practice. Transfusion Medicine 1998;8:281-02.

e. Arce MA, Thompson ES, Wagner S, Coyne KE, Ferdman BA, Lublin DM. Molecular cloning of RhD cDNA-derived from a gene present in RhD-positive, but not RhD-negative individuals. Blood 1993;82:651-5.
f. Avent ND, Reid ME. The Rh blood group system: a review. Blood 2000; 95:375-387.
g. Raymaeker M, Smets R, Maes B, Cartuyvels R. Checklist for optimization and validation of real-time PCR assays. Journal Clinical Laboratory Analysis 2009;23:145-151.
h. Udvardi MK, Czechowski T, Scheible WR. Eleven golden rules of quantitative real-time PCR. The Plant Cell 2008;20:1736-1737.
i. Finning KM, Martin PG, Soothill PW, Avent ND. Prediction of fetal D status from maternal plasma: introduction of a new noninvasive fetal RHD genotypig service. 2002;42:1079-1085.

j. Bustin SA, Benes V, Garson JA, Hellemans J, Huggett J, Kubista M, Mueller R, Nolan T, Pfaffl MW, Shipley GL Vandesompele J, Wittwer CT. The MIQE Guidelines: Minimum Information for publication of Quantitative real-time PCR Experiments. 2009;55:611-622.
k. BIO-RAD CFX Manager Software Protocol Quick Guide: www.bio-rad.com/amplification.
