# Supplementary material for: Transitioning a multiethnic donor pool from serologic D-negative to molecularly RHD-negative at a hospital-based blood donor service
Source: J Transl Med. 2025 Jun 19;23:686. doi: 10.1186/s12967-025-06716-8 (PMC12180192; doi:10.1186/s12967-025-06716-8)
Supplement: Supplementary file 2 — Additional file 2. Supplementary Table S1. Molecular screening of serologic D-negative donors for the RHD gene in 58 studies. A complete list of publications from 2001 to 2024 has been compiled documenting the types of RHD screening and the total number of donors screened worldwide in clinical studies over the past 25 years. [file 12967_2025_6716_MOESM2_ESM.pdf]

Supplementary Table S1. Molecular screening of serologic D-negative donors for the *RHD* gene in 58 studies

Population	Year	Screening method	*RHD* sequence tested	Serologic D-negative donors						Comments and Reference
				All (n)	*RHD* sequence positive (n)	%	C+/E+ (n)	*RHD* sequence positive (n)	%	
Japan	1997	PCR-SSP	intron 4, exon 10	130	38	29.2	103	38	36.9	1
Japan	1997	PCR-SSP	exons 4, 7, 10	306	102	33.3	247	102	41.3	2
Taiwan	1998	PCR-SSP	intron 4, exons 7, 10	230	85	37.0	na	na	na	3
China	2000	PCR-SSP	intron 4, exons 3 to 7, 9, 10	87	35	40.2	44	35	79.5	4
Germany	2001	PCR-SSP	5′-UTR, intron 4, exons 7, 10	8,442	50	0.6	754	48	6.4	South-West region[5]
Brazil	2002	PCR-SSP	intron 4, exon 10	206	27	13.1	na	na	na	6
China	2002	PCR-SSP	exons 1 to 10	102	38	37.3	na	na	na	7
China	2003	PCR-SSP	intron 4, exons 2 to 7, 9, 10	131	48	36.6	71	48	67.6	8
Taiwan	2003	PCR-RFLP	introns 1, 2, 4, 8, exons 1, 3 to 7, 9, 10	204	54	26.5	56	54	96.4	9
Taiwan	2003	PCR-SSP	exons 3, 4, 5, 7, 9	156	48	30.8	79	47	59.5	10
Taiwan	2004	PCR-SSP	exons 4, 5, 7, 9, 10	294	109	37.1	132	109	82.6	11
Europe[a]	2005	PCR-SSP	5′-UTR, exons 3, 10	na	na	na	1,700	89	5.2	12
China	2005	real-time PCR	exon 7	74	28	37.8	na	na	na	13
Korea	2005	PCR-SSP	intron 4, exons 3, 5, 7, 10	264	68	25.8	107	68	63.5	14
China	2006	PCR-SSP	exons 3 to 7, 9	515	79	15.3	235	79	33.6	15
Korea	2006	PCR-SSP	promoter, intron 4, exons 7 and 10	126	32	25.4	54	32	59.2	16
Austria	2007	real-time PCR	exons 4, 7, 10	2,427	3	0.1	na	na	na	17
Germany	2009	PCR-SSP	intron 4	46,133	96	0.2	3,080	81	2.6	18
China	2009	PCR-SSP	exons 3 to 7, 9	1,585	348	22.0	769	347	45.1	19
Austria	2009	real-time PCR	exons 4, 7, 10	23,330	94	0.4	1,934	62	3.2	20
Congo	2009	PCR-SSP	exons 4, 5, 7, 10	110	51	46.4	na	na	na	21
Denmark	2010	PCR-SSP	exon 10	na	na	na	233	7	4.4	22
Canada	2010	PCR-SSP	exons 4, 5	3,980	13	0.3	na	na	na	23
Denmark	2011	real-time PCR	exons 5, 7, 10	5,058	13	0.3	na	na	na	24
Italy	2011	PCR-SSP	introns 4 and 7, exon 7	na	na	na	235	12	5.1	25
Brazil	2012	PCR-SSP	intron 4, exon 10	239	22	9.2	18	11	61.1	26

Brazil	2012	PCR-SSP	intron 4, exon 7	2,450	101	4.1	na	na	na	27
Tunisia	2012	PCR-SSP	exon 10	448	11	2.5	45	10	22.2	28
Brazil	2012	real-time PCR	exons 7, 10	203	25	12.3	na	na	na	29
China	2012	PCR-SSP	5′-UTR, intron 4, exons 7, 10	155	35	22.6	na	na	na	30
Germany	2012	real-time PCR	exon 7	46,756	122	0.3	na	na	na	Northern region[31]
Poland	2013	real-time PCR	intron 4, exons 7, 10	31,200	63	0.2	na	na	na	32
Tunisia	2013	PCR-SSP	exons 3 to 7, 9	223	5	2.2	na	na	na	33
China	2013	PCR-SSP	exons 1, 4, 5, 6, 8, 9	2,493	808	32.4	1,093	723	66.1	34
Switzerland	2014	PCR-SSP	exons 3, 5, 10	25,370	120	0.5	na	na	na	35
Switzerland	2014	PCR-SSP	exons 3, 5, 10	26,243	65	0.2	na	na	na	36
Argentina	2014	PCR-SSP	intron 4 and 3′-UTR	1,314	27	2.1	114	19	16.7	37
Australia	2014	real-time PCR	exons 4, 5, 10	na	na	na	2,027	44	2.2	38
Tunisia	2014	PCR-SSP	exon 10	400	10	2.5	38	7	18.4	39
China	2014	PCR-SSP	exons 1 to 10	165	41	24.8	na	na	na	40
Brazil	2014	PCR-SSP	intron 4, exon 10	na	na	na	520	18	3.5	41
Tunisia	2014	PCR-SSP	exon 10	na	na	na	100	25	25.0	42
Netherlands	2016	MLPA	5′-UTR, exons 1 to 10	37,782	270	0.7	na	na	na	43
India	2018	QMPSF	exons 1 to 10	171	53	31	171	53	31	44
Brazil	2019	PCR-SSP	intron 4, exon 7	1,403	102	7.3	517	261	50.5	45
Iran	2019	real-time PCR	exons 5, 7, 10	200	2	1.0	16	2	12.5	46
United States	2019	ASOH	exons 1 to 10	1,163	48	4.1	81	5	6.2	BioArray *RHD* BeadChip[47]
Thailand	2019	QMPSF	exons 1 to 10	200	50	25	100	50	50	48
Iran	2021	real-time PCR	exons 5, 7, 10	200	2	1.0	na	na	na	49
Korea	2021	PCR-SSP	promoter, exons 4, 7, 10	na	na	na	177	109	61.6	50
China	2022	PCR-SSP	exon 10	104	26	25.0	na	na	na	51
India	2022	PCR-SSP	intron 4, exon 4, exon 7	200	5	2.5	190	4	2.1	52
Thailand	2022	QMPSF	exons 1 to 10	1270	307	24.2	598	305	51	53
Thailand	2022	PCR-SSP	exon 4	191	59	30.9	na	na	na	54
Congo	2024	PCR-SSP	exons 4, 5, 7, 10	59	53	89.8	na	na	na	55
BH[b]	2024	real-time PCR	exons 3, 5, 10	12,827	40	0.31	481	40	8.32	RBC-FluoGene[56]
Brazil	2024	PCR-SSP	intron 4, exon 7	648	17	2.6	na	na	na	57
Saudi Arabia	2024	PCR-SSP	exons 3, 4, 7	66	0	na	4	0	na	58

United States	2024	real-time PCR	intron 4, exons 5, 7	2,254	42	1.9	115	10	8.69	this study
Total				290,287	3,690	na	16,238	2,954	na	

a Multicenter study including Austria, Germany, Slovenia, Switzerland and Russia
b Bosnia and Herzegovina
na, not available or not applicable
PCR=polymerase chain reaction; PCR-SSP=PCR with sequence-specific primers; UTR=untranslated region; PCR-RFLP=PCR-restriction fragment length polymorphism; MLPA=multiplex ligation-dependent probe amplification; ASOH=allele specific oligonucleotide hybridization; QMPSF=quantitative multiplex polymerase chain reaction (PCR) of short fluorescent fragments

References

1. Okuda H, Kawano M, Iwamoto S, Tanaka M, Seno T, Okubo Y, et al. The *RHD* gene is highly detectable in RhD-negative Japanese donors. J Clin Invest 1997;100:373-9.
2. Fukumori Y, Hori Y, Ohnoki S, Nagao N, Shibata H, Okubo Y, et al. Further analysis of D_{el} (D-elute) using polymerase chain reaction (PCR) with *RHD* gene-specific primers. Transfus Med 1997;7:227-31.
3. Sun CF, Chou CS, Lai NC, Wang WT. *RHD* gene polymorphisms among RhD-negative Chinese in Taiwan. Vox Sang 1998;75:52-7.
4. Lan JC, Chen Q, Wu DL, Ding H, Pong DB, Zhao T. Genetic polymorphism of RhD-negative associated haplotypes in the Chinese. J Hum Genet 2000;45:224-7.
5. Wagner FF, Frohmajer A, Flegel WA. *RHD* positive haplotypes in D negative Europeans. BMC Genet 2001;2:10.
6. Rodrigues A, Rios M, Pellegrino J, Jr., Costa FF, Castilho L. Presence of the *RHD* pseudogene and the hybrid *RHD-CE-D*^{s} gene in Brazilians with the D-negative phenotype. Braz J Med Biol Res 2002;35:767-73.
7. Shao CP, Maas JH, Su YQ, Köhler M, Legler TJ. Molecular background of Rh D-positive, D-negative, D_{el} and weak D phenotypes in Chinese. Vox Sang 2002;83:156-61.
8. Xu Q, Zhang J, Wang Q, Zhang S, Si G. *RHD* gene polymorphism among RhD-negative Han Chinese. Chin Med J (Engl) 2003;116:1539-43.
9. Peng CT, Shih MC, Liu TC, Lin IL, Jaung SJ, Chang JG. Molecular basis for the RhD negative phenotype in Chinese. Int J Mol Med 2003;11:515-21.
10. Lee YL, Chiou HL, Hu SN, Wang L. Analysis of *RHD* genes in Taiwanese RhD-negative donors by the multiplex PCR method. J Clin Lab Anal 2003;17:80-4.
11. Chen JC, Lin TM, Chen YL, Wang YH, Jin YT, Yue CT. *RHD 1227A* is an important genetic marker for RhD_{el} individuals. Am J Clin Pathol 2004;122:193-8.
12. Gassner C, Doescher A, Drnovsek TD, Rozman P, Eicher NI, Legler TJ, et al. Presence of *RHD* in serologically D-, C/E+ individuals: a European multicenter study. Transfusion 2005;45:527-38.
13. Xu Q, Grootkerk-Tax MG, Maaskant-van Wijk PA, van der Schoot CE. Systemic analysis and zygosity determination of the *RHD* gene in a D-negative Chinese Han population reveals a novel D-negative *RHD* gene. Vox Sang 2005;88:35-40.
14. Kim JY, Kim SY, Kim CA, Yon GS, Park SS. Molecular characterization of D- Korean persons: development of a diagnostic strategy. Transfusion 2005;45:345-52.
15. Li Q, Ye LY, Guo ZH, Qian M, Zhu ZY. [Study on the molecular background of Del phenotype in Chinese population]. Zhonghua Yi Xue Yi Chuan Xue Za Zhi 2006;23:486-91.
16. Luettringhaus TA, Cho D, Ryang DW, Flegel WA. An easy *RHD* genotyping strategy for D- East Asian persons applied to Korean blood donors. Transfusion 2006;46:2128-37.
17. Polin H, Danzer M, Hofer K, Gassner W, Gabriel C. Effective molecular *RHD* typing strategy for blood donations. Transfusion 2007;47:1350-5.
18. Flegel WA, von Zabern I, Wagner FF. Six years' experience performing *RHD* genotyping to confirm D- red blood cell units in Germany for preventing anti-D immunizations. Transfusion 2009;49:465-71.
19. Li Q, Hou L, Guo ZH, Ye LY, Yue DQ, Zhu ZY. Molecular basis of the *RHD* gene in blood donors with DEL phenotypes in Shanghai. Vox Sang 2009;97:139-46.
20. Polin H, Danzer M, Gaszner W, Broda D, St-Louis M, Pröll J, et al. Identification of *RHD* alleles with the potential of anti-D immunization among seemingly D- blood donors in Upper Austria. Transfusion 2009;49:676-81.
21. Touinssi M, Chapel-Fernandes S, Granier T, Bokilo A, Bailly P, Chiaroni J. Molecular analysis of inactive and active *RHD* alleles in native Congolese cohorts. Transfusion 2009;49:1353-60.

22. Christiansen M, Sørensen BS, Grunnet N. *RHD* positive among C/E+ and D- blood donors in Denmark. Transfusion 2010;50:1460-4.
23. St-Louis M, Perreault J, Lavoie J, Émond J, St-Laurent J, Long A, et al. [Genotyping of 21,000 blood donors in Quebec and *RHD* analysis]. Transfus Clin Biol 2010;17:242-8.
24. Krog GR, Clausen FB, Berkowicz A, Jørgensen L, Rieneck K, Nielsen LK, et al. Is current serologic RhD typing of blood donors sufficient for avoiding immunization of recipients? Transfusion 2011;51:2278-85.
25. Londero D, Fiorino M, Miotti V, de Angelis V. Molecular RH blood group typing of serologically D-/CE+ donors: the use of a polymerase chain reaction-sequence-specific primer test kit with pooled samples. Immunohematology 2011;27:25-8.
26. Cruz BR, Chiba AK, Moritz E, Bordin JO. *RHD* alleles in Brazilian blood donors with weak D or D-negative phenotypes. Transfus Med 2012;22:84-9.
27. Mota M, Dezan M, Valgueiro MC, Sakashita AM, Kutner JM, Castilho L. *RHD* allelic identification among D-Brazilian blood donors as a routine test using pools of DNA. J Clin Lab Anal 2012;26:104-8.
28. Moussa H, Tsochandaridis M, Chakroun T, Jridi S, Abdelneji B, Hmida S, et al. Molecular background of D-negative phenotype in the Tunisian population. Transfus Med 2012;22:192-8.
29. Szulman A, Nardozza LM, Barreto JA, Araujo Júnior E, Moron AF. Investigation of pseudogenes *RHDΨ* and *RHD-CE-D* hybrid gene in D-negative blood donors by the real time PCR method. Transfus Apher Sci 2012;47:289-93.
30. Chen Q, Li M, Li M, Lu XS, Lü R, Sun J, et al. Molecular basis of weak D and DEL in Han population in Anhui Province, China. Chin Med J (Engl) 2012;125:3251-5.
31. Wagner FF MI, Bittner R, Döscher A. *RHD* PCR of blood donors in Northern Germany: use of adsorption/elution to determine D antigen status (abstract 3C-S8–04). Vox Sang 2012;103:15.
32. Orzińska A, Guz K, Polin H, Pelc-Kłopotowska M, Bednarz J, Gieleżyńska A, et al. *RHD* variants in Polish blood donors routinely typed as D-. Transfusion 2013;53:2945-53.
33. Ouchari M, Jemni-Yaacoub S, Chakroun T, Abdelkefi S, Houissa B, Hmida S. *RHD* alleles in the Tunisian population. Asian J Transfus Sci 2013;7:119-24.
34. Ye SH, Wu DZ, Wang MN, Wu XY, Xu HG, Xu H, et al. A comprehensive investigation of *RHD* polymorphisms in the Chinese Han population in Xi'an. Blood Transfus 2014;12:396-404.
35. Crottet SL, Henny C, Meyer S, Still F, Stolz M, Gottschalk J, et al. Implementation of a mandatory donor RHD screening in Switzerland. Transfus Apher Sci 2014;50:169-74.
36. Gowland P, Gassner C, Hustinx H, Stolz M, Gottschalk J, Tissot JD, et al. Molecular *RHD* screening of RhD negative donors can replace standard serological testing for RhD negative donors. Transfus Apher Sci 2014;50:163-8.
37. Trucco Boggione C, Luján Brajovich ME, Tarragó M, Mattaloni SM, Biondi CS, Muñiz-Díaz E, et al. Molecular structures identified in serologically D-samples of an admixed population. Transfusion 2014;54:2456-62.
38. Scott SA, Nagl L, Tilley L, Liew YW, Condon J, Flower R, et al. The *RHD(1227G>A)* DEL-associated allele is the most prevalent *DEL* allele in Australian D-blood donors with C+ and/or E+ phenotypes. Transfusion 2014;54:2931-40.
39. Sassi A, Ouchari M, Houissa B, Romdhane H, Abdelkefi S, Chakroun T, et al. *RHD* genotyping and its implication in transfusion practice. Transfus Apher Sci 2014;51:59-63.
40. Gu J, Wang XD, Shao CP, Wang J, Sun AY, Huang LH, et al. Molecular basis of DEL phenotype in the Chinese population. BMC Med Genet 2014;15:54.
41. Costa S, Martin F, Chiba A, Langhi D, Jr., Chiattone C, Bordin J. *RHD* alleles and D antigen density among serologically D- C+ Brazilian blood donors. Transfus Med 2014;24:60-1.

42. Moussa H, Tsochandaridis M, Kacem N, Chakroun T, Abdelkefi S, Gabert J, et al. *RHD* positive among C/E+ and D-negative blood donors in Tunisia. Transfus Clin Biol 2014;21:320-3.
43. Stegmann TC, Veldhuisen B, Bijman R, Thurik FF, Bossers B, Cheroutre G, et al. Frequency and characterization of known and novel *RHD* variant alleles in 37,782 Dutch D-negative pregnant women. Br J Haematol 2016;173:469-79.
44. Kulkarni SS, Gogri H, Parchure D, Mishra G, Ghosh K, Rajadhyaksha S, et al. RHD-Positive Alleles among D- C/E+ Individuals from India. Transfus Med Hemother 2018;45:173-7.
45. de Paula Vendrame TA, Prisco Arnoni C, Guilhem Muniz J, de Medeiros Person R, Pereira Cortez AJ, Roche Moreira Latini F, et al. Characterization of *RHD* alleles present in serologically RHD-negative donors determined by a sensitive microplate technique. Vox Sang 2019;114:869-75.
46. Khosroshahi BN, Oodi A, Namjou S, Gholamali T, Amirizadeh N. *RHD* genotyping by molecular analysis of hybrid rhesus box in RhD-negative blood donors from Iran. Indian J Hematol Blood Transfus 2019;35:119-24.
47. Perez-Alvarez I, Hayes C, Hailemariam T, Shin E, Hutchinson T, Klapper E. *RHD* genotyping of serologic RhD-negative blood donors in a hospital-based blood donor center. Transfusion 2019;59:2422-8.
48. Thongbut J, Raud L, Férec C, Promwong C, Nuchnoi P, Fichou Y. Comprehensive Molecular Analysis of Serologically D-Negative and Weak/Partial D Phenotype in Thai Blood Donors. Transfus Med Hemother 2020;47:54-60.
49. Sadeghi-Bojd Y, Amirizadeh N, Oodi A. *RHD* genotyping of Rh-negative and weak D phenotype among blood donors in Southeast Iran. Int J Hematol Oncol Stem Cell Res 2021;15:213-20.
50. Kim T, Park Y, Shin L, Jung YS, Youn M, Kim Y. The experience of *RHD* genotyping in D-negative blood donors. Korean J Blood Transfus 2021;32:91-101.
51. Ren Q, Cao L, Xia Y, Xu H, Xie Y, Mi Z, et al. Genetic background of RhD negative blood donors. Zhongguo shuxue zazhi 2022;35:1014-6.
52. Khetan D, Shukla JS, Chaudhary RK. Molecular basis of RhD-negative phenotype in North Indian blood donor population. Indian J Med Res 2022;155:286-92.
53. Nuchnoi P, Thongbut J, Bénech C, Kupatawintu P, Chaiwanichsiri D, Férec C, et al. Serologically D-negative blood donors in Thailand: molecular variants and diagnostic strategy. Blood Transfus 2023;21:209-17.
54. Simtong P, Phothi R, Puapairoj C, Leelayuwat C, Romphruk AV. *RHD* 1227 A and hybrid Rhesus box analysis in Thai RhD+ and RhD- blood donors: Prevalence, *RHD* zygosity, and molecular screening. Transfus Apher Sci 2022;61:103496.
55. Fouti L, Mpelle L, Ondzia F, Senda L, Bikoue A, Boumba A, et al. Molecular characterization of *RHD* in Rh-negative blood donors in Congo Brazzaville. J. Biosci. Med. 2024;12:299-309.
56. Lilić M, Guzijan G, Jovanović Srzentić S. Rare occurrence of *RHD* null alleles with Del expression among serologically D-negative blood donors. Scripta Medica 2024;55:307-15.
57. Rodrigues ES, Santos FLS, de Paula Vendrame TA, Cuter TB, Romagnoli A, Mendes V, et al. Rh D-positive genotypes in Brazilian blood donors with D-negative phenotype. Ann Blood 2024;9:19-22.
58. Alalshaikh MA, Alsughayir AH, Alsaif AS, Ababtain SA, Aloyouni SY, Aldilaijan KE, et al. Molecular background of RhD-positive and RhD-negative phenotypes in a Saudi population. Saudi J Med Med Sci 2024;12:210-5.
